# ASO Author Reflections: Analysis of Treatment Strategies and Outcomes in Malignant Peritoneal Mesothelioma: Insights from a Multi-center Study

**DOI:** 10.1245/s10434-024-15653-7

**Published:** 2024-06-25

**Authors:** Serkan Yaşar

**Affiliations:** Department of Medical Oncology, Hacettepe University Oncology Institute, Ankara, Turkey

## Past

Standardization of treatment approaches to patient selection in the treatment of malignant peritoneal mesothelioma (MPeM) has not been achieved. Clear answers could not be found for questions such as which patients should undergo surgery, which patients should undergo surgery and hyperthermic intraperitoneal chemotherapy (HIPEC), and in which cases systemic treatment should be applied instead of surgery. Additionally, experience with perioperative treatment was quite limited.

## Present

The course of the disease was significantly unfavorable due to limited experience in the application of multimodal treatments, surgical techniques, and appropriate patient selection for surgery, but recently with the development of multimodal treatments combining systemic chemotherapies such as cytoreductive surgery (CRS) and HIPEC, survival has increased significantly.^1^ Some studies have shown that appropriate patient selection for CRS-HIPEC is very important, and that with a successful procedure, overall survival can extend from 1 year to a period of 34 to 92 months.^2–4^ The most important prognostic factor in patients suitable for surgery is complete or near-complete cytoreduction. With clinicians expecting good cytoreduction when deciding on surgery, survival is significantly prolonged, as confirmed in this study. The results showed that the prognosis of the group with a lower cytoreduction score (CC score) was better.^5^ Additionally, the study showed that cytotoxic chemotherapies still are the best option for patients who are not candidates for curative surgery. If the patient is not suitable for surgery and chemotherapy is applied, the patient experiences more benefit. Otherwise, a high postoperative CC score may cause detrimental effects. In addition, factors such as age, presence of ascites, pre-treatment Eastern Cooperative Oncology Group (ECOG) Performance Status, histologic type, gender, and asbestos exposure also were evaluated, and their effects on prognosis were examined. The findings showed that a broad perspective and multidisciplinary study is required when clinicians are making treatment decisions.^6^

## Future

Thanks to recent developments and accumulated experience, MPeM management currently is in a better situation than in the past. However, it is accepted that areas for improvement remain in both surgical and non-surgical patient groups. The authors understand that surgery always seems to be the primary treatment, but clinicians do not have enough options in case of surgical failure or disease spread. New and effective agents are needed, especially for use in the perioperative and metastatic site. The lack of response to traditional chemotherapeutic agents led researchers to discover new agents. Ongoing studies show that treatment options will increase. In light of these developments, well-selected patients, experienced surgeons, and the use of old and new agents, especially in the perioperative period, clinicians can hope for better results.
